# Is there a progression‐free survival benefit of first‐line crizotinib versus standard chemotherapy and second‐line crizotinib in *ALK*‐positive advanced lung adenocarcinoma? A retrospective study of Chinese patients

**DOI:** 10.1002/cam4.659

**Published:** 2016-02-16

**Authors:** Shaohua Cui, Yizhuo Zhao, Lili Dong, Aiqin Gu, Liwen Xiong, Jialin Qian, Wei Zhang, Yanjie Niu, Feng Pan, Liyan Jiang

**Affiliations:** ^1^ Department of Pulmonary MedicineShanghai Chest HospitalShanghai Jiao Tong UniversityShanghaiChina

**Keywords:** Anaplastic lymphoma kinase, crizotinib, first‐line chemotherapy, lung adenocarcinoma, progression‐free survival

## Abstract

Although crizotinib has demonstrated promising efficacy and acceptable toxicity in patients with advanced non‐small cell lung cancer (NSCLC), the available evidence in Chinese populations is currently limited. This study compared the progression‐free survival (PFS) of Chinese patients with anaplastic lymphoma kinase (*ALK*)‐positive, advanced lung adenocarcinoma who received first‐line crizotinib therapy with that of patients who received first‐line standard chemotherapy, and also the PFS benefit of first‐line versus second‐line crizotinib treatment. Data on 80 patients with *ALK*‐positive, advanced lung adenocarcinoma who received crizotinib or standard chemotherapy as first‐line treatments between June 2013 and December 2014 were retrospectively collected; 26 of the patients received crizotinib as second‐line therapy after progressive disease (PD) occurred on first‐line chemotherapy. Tumor responses were assessed using Response Evaluation Criteria in Solid Tumors (RECIST), version 1.1. The median PFS was 13.3 months (95% CI: 6.5–20.0 months) in patients who received first‐line crizotinib as compared with 5.4 months (95% CI: 4.4–6.5 months) in patients who received first‐line standard chemotherapy (adjusted hazard ratio for progression or death with crizotinib, 0.20; 95% CI: 0.11–0.36; *P *< 0.001). In patients who received second‐line crizotinib therapy, the median PFS was 9.9 months (95% CI: 6.4–13.4 months). The difference between first‐line and second‐line crizotinib treatment was not statistically significant (adjusted hazard ratio for progression, 0.56; 95% CI: 0.29–1.11; *P *= 0.092). Thus, there was a significant PFS benefit of first‐line crizotinib versus first‐line standard chemotherapy in Chinese patients with *ALK*‐positive lung adenocarcinoma. Additionally, crizotinib showed promising efficacy in patients who received it as second‐line therapy after PD had occurred on first‐line chemotherapy.

## Introduction

Anaplastic lymphoma kinase (*ALK*) is a tyrosine kinase target recently validated in a subset of non‐small cell lung cancers (NSCLC) 1. Rearrangements of the *ALK* gene, which most often consists of a chromosome 2 inversion that leads to fusion with the echinoderm microtubule‐associated protein‐like 4 (*EML4*) gene, are found in approximately 2–7% of patients with NSCLC 1, 2, 3, 4. *ALK*‐positivity may define a molecular subgroup of NSCLC, since this mutation generally occurs in younger patients who have never smoked and have an adenocarcinoma histology 5, 6, 7. However, a recent large‐scale study of Chinese patients indicated that a younger age at diagnosis was the only independent factor associated with *EML4‐ALK* rearrangement 8.

Crizotinib, an ATP‐competitive, oral, small‐molecule tyrosine kinase inhibitor that targets *ALK*, mesenchymal–epithelial transition (*MET*), and *ROS1* 9, 10, has demonstrated promising efficacy and acceptable toxicity in many studies of patients with advanced NSCLC 3, 11, 12, 13, 14. Phase 1 and 2 studies of crizotinib treatment have shown that the progression‐free survival (PFS) of patients with *ALK*‐positive NSCLC was 7–10 months, and the objective response rate (ORR) was approximately 60% 3, 11, 12. Recently, a phase 3 study comparing crizotinib and standard chemotherapy showed that crizotinib was superior to pemetrexed or docetaxel in previously treated, *ALK*‐positive, advanced NSCLC. The median PFS in the crizotinib and chemotherapy groups was 7.7 and 3.0 months, respectively (*P *< 0.001), and the ORR was 65% and 20%, respectively (*P *< 0.001) 13. More recently, in a phase 3 study (PROFILE 1014) comparing first‐line crizotinib with first‐line pemetrexed plus platinum chemotherapy, the PFS and ORR of the crizotinib group were significantly superior to those of the chemotherapy group (median PFS, 10.9 vs. 7.0 months, respectively (*P *< 0.001); ORR, 74% vs. 45%, respectively (*P *< 0.001)) 14. The most common adverse events with crizotinib reported in these studies were transient visual disturbance and gastrointestinal reactions, whereas fatigue, alopecia, and gastrointestinal disturbances were common with standard chemotherapy 13, 14. These studies indicate that crizotinib is superior to chemotherapy in the treatment of *ALK*‐positive NSCLC. Currently, however, there is only limited evidence of the efficacy and tolerability of crizotinib in Chinese patients with *ALK*‐positive lung adenocarcinoma.

This study was mainly conducted to compare the PFS of Chinese patients with *ALK*‐positive lung adenocarcinoma who received first‐line crizotinib therapy with that of patients who received first‐line standard chemotherapy. In addition, we also studied whether there is a PFS benefit of first‐line versus second‐line crizotinib therapy in these patients.

## Patients and Methods

### Patients

Data on a total of 80 patients with *ALK*‐positive (as determined by Ventana immunohistochemistry initially and confirmed by fluorescence *in situ* hybridization (FISH)) advanced lung adenocarcinoma, who received either crizotinib or standard chemotherapy as first‐line treatments between June 1, 2013 and December 31, 2014 at Shanghai Chest Hospital, Shanghai Jiao Tong University, were retrospectively collected and analyzed; 30 patients received first‐line crizotinib therapy, while 50 patients received first‐line platinum‐based chemotherapy regimens. All patients were histologically diagnosed and staged as having clinically advanced (stage IV or stage IIIB) lung adenocarcinoma prior to treatment.

Before initiation of therapy, all patients were evaluated by computed tomography (CT) of the thorax, an electrocardiogram (ECG), enhanced magnetic resonance imaging (MRI) of the brain, a whole body bone scan, and abdominal ultrasound. Additionally, routine hematology and biochemistry tests, coagulation tests, and urinalyses were also performed before treatment, and a medical history was taken from each patient. Patients who received systemic therapy (including targeted therapy and chemotherapy) before June 1, 2013 or who had symptomatic brain metastases or an Eastern Cooperative Oncology Group performance status (ECOG PS) of more than 2 were not enrolled. Age, gender, smoking status, clinical stage, toxicity during treatment, the response data, and PFS data were collected for all patients. In addition, data on 26 patients who received crizotinib as second‐line treatment after disease progression on first‐line chemotherapy were also analyzed.

The study was approved by Ethics Committee of Shanghai Chest Hospital, Shanghai Jiao Tong University, Shanghai.

### *ALK* detection

Tumor samples obtained by either diagnostic or surgical procedures were used for *ALK* mutation detection. We used immunohistochemical analysis which was conducted with the monoclonal antibody D5F3 (Ventana Medical Systems, Tucson, AZ) to screen *ALK*‐positive cases. Fluorescence *in situ* hybridization was then used to confirm the outcomes of immunohistochemical analysis, and the positive cutoff value of FISH was defined as 15%.

### Treatment, response evaluation, and follow‐up

In the patients who received crizotinib, the dosage administered was 250 mg orally twice daily in 28‐day cycles. The tumor response in this group was assessed after the first cycle of treatment and subsequently after every 2 cycles. Patients continued to receive crizotinib treatment as long as they did not have progressive disease (PD) or intolerable adverse effects.

In the patients who received standard chemotherapy, either pemetrexed (500 mg/m^2^ of body surface area), docetaxel (75 mg/m^2^), or gemcitabine (1250 mg/m^2^ on days 1 and 8) plus either cisplatin (75 mg/m^2^) or carboplatin (target area under the curve of 5–6 mg/mL per min) were administered intravenously in 21‐day cycles. The tumor response in this group was assessed every 2 cycles.

Tumor responses were assessed using the Response Evaluation Criteria in Solid Tumors (RECIST; http://www.eortc.org/news/recist-news-and-updates/), version 1.1. During follow‐up, CT scans of the thorax, enhanced MRI of the brain, whole body bone scans, and abdominal ultrasound were used to assess the response to crizotinib and standard chemotherapy. Response to treatments was reported as either a complete response (CR), partial response (PR), stable disease (SD), or PD. Patients continued to receive crizotinib treatment even when they had been assessed as PD by RECIST criteria as we believed these patients would acquire clinical benefit from the drug. The cutoff date for the study was December 31, 2015.

### Assessment of tolerability

Tolerability was assessed at least twice per treatment cycle by the occurrence of adverse events, ECG findings, routine hematology and biochemistry tests, coagulation tests, and urinalyses. All toxicities were summarized according to the National Cancer Institute's Common Terminology Criteria for Adverse Events (NCI CTCAE), version 3.0. Tolerability data were collected not only from patients who received crizotinib or standard chemotherapy as first‐line treatment, but also from those who received crizotinib as second‐line treatment after the occurrence of PD on first‐line chemotherapy.

### Statistical analysis

Two‐sided Fisher's exact tests were used for analyzing patients' basic characteristics in different groups, and comparing the ORR and disease control rates (DCR) between different treatment groups. PFS was calculated as the time from the date treatment was first administered until the date of objective PD, according to RECIST, or death from any cause. The Kaplan–Meier method was applied to estimate PFS, and two‐sided log‐rank tests were applied to compare differences between the treatment groups. A Cox regression model was used to estimate hazard ratios. *P* values less than 0.05 were considered statistically significant. Statistical analyses was performed using SPSS^®^ software, version 13.0 (SPSS Inc., Chicago, IL).

## Results

### Patient characteristics and treatments

**Figure 1:**
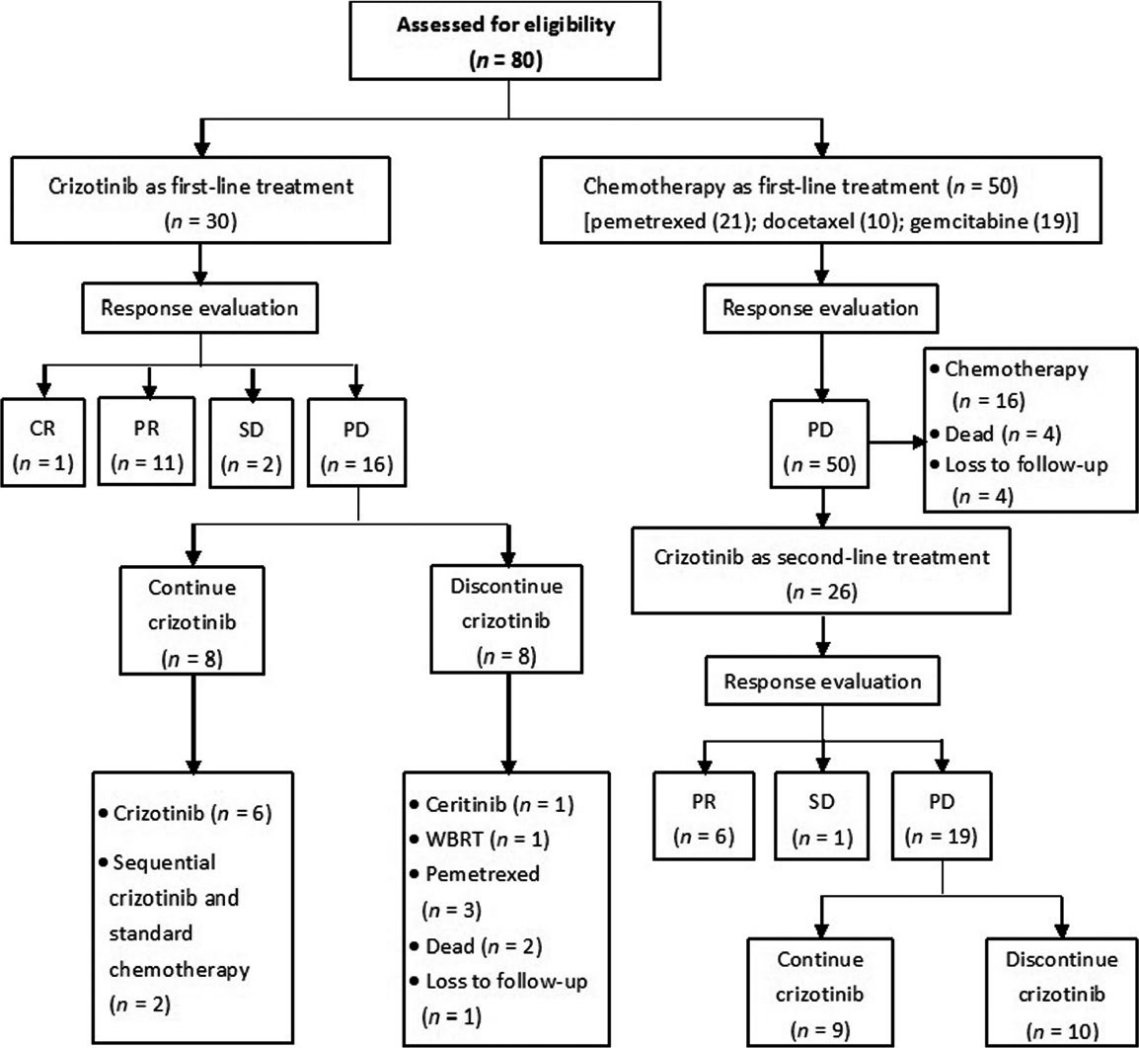
Overview of treatment of the 80 anaplastic lymphoma kinase (*ALK*)‐positive lung adenocarcinoma patients. CR, complete response; PD, progressive disease; PR, partial response; SD, stable disease; WBRT, whole‐brain radiotherapy.

Treatment of the 80 *ALK*‐positive, lung adenocarcinoma patients analyzed in the study is summarized in Figure 1, and the patients' demographic and clinicopathologic characteristics are shown in Table 1. The patients tended to be young (mean age 54 years, range 26–83 years) and never or light smokers (≤ 10 pack‐year). Seventeen patients (21.3%) had a history of radical surgery before treatment with crizotinib or chemotherapy. The numbers of patients who received crizotinib and standard chemotherapy as first‐line therapy were 30 (37.5%) and 50 (62.5%), respectively.

**Table 1:** Demographic and clinicopathologic characteristics of 80 anaplastic lymphoma kinase (*ALK)*‐positive patients who received crizotinib or chemotherapy as first‐line treatment

Characteristic	Total *N *= 80*n* (%)	Crizotinib *N *= 30*n* (%)	Chemotherapy *N *= 50*n* (%)	*P*‐Value
Age, years				
Mean	54	58	52	
Range	26–83	37–83	26–72	
Age distribution				
<60 years	49 (61)	17 (57)	32 (64)	0.636
≥60 years	31 (39)	13 (43)	18 (36)	
Gender				
Male	38 (48)	15 (50)	23 (46)	0.819
Female	42 (52)	15 (50)	27 (54)	
ECOG PS				
0	22 (28)	8 (27)	14 (28)	1.000
1	55 (69)	21 (70)	34 (68)	
2	3 (3)	1 (3)	2 (4)	
Radical surgery history				
Yes	17 (21)	7 (23)	10 (20)	0.781
No	63 (79)	23 (77)	40 (80)	
Smoking history				
Never	59 (74)	23 (77)	36 (72)	0.625
≤10 pack‐year	10 (13)	1 (3)	5 (10)	
>10 pack‐year	11 (13)	6 (20)	9 (18)	
Clinical stage				
IIIB	6 (8)	1 (3)	5 (10)	0.402
IV	74 (92)	29 (97)	45 (90)	

ECOG PS, Eastern cooperative oncology group performance status.

In the patients who received crizotinib as first‐line treatment, 16 (53.3%) were assessed as having PD according to RECIST at the cutoff date, and eight of these patients (50.0%) discontinued crizotinib treatment. One patient (6.3%) was subsequently treated with ceritinib (LDK 378), one (6.3%) received whole‐brain radiotherapy for a brain metastasis, three (18.8%) received pemetrexed plus platinum as second‐line chemotherapy, two patients (12.5%) died during the subsequent follow‐up, and one (6.3%) was lost to follow‐up.

In the patients who received chemotherapy as first‐line treatment, all were assessed as having PD at the study cutoff date. Twenty‐six of the 50 patients (52.0%) were treated with crizotinib as a second‐line regimen after PD had been defined (four of whom died after PD occurred on second‐line crizotinib therapy) while 16 (32.0%) received other chemotherapy regimens as second‐line treatment. One patient (2.0%) died during first‐line chemotherapy for reasons associated with disease progression, three (6.0%) died after PD occurred on first‐line treatment, and four (8.0%) were lost to follow‐up. Of the 26 patients who received second‐line crizotinib after the occurrence of PD on first‐line chemotherapy, 19 patients (73.1%) had occurred PD at the study cutoff date.

Seventeen of the 56 patients (30.4%) who received crizotinib as first‐line or second‐line treatment continued to take crizotinib after PD was defined according to RECIST as we believed these patients would acquire clinical benefit from the drug.

The demographic and clinicopathologic characteristics of the 56 patients who received crizotinib as first‐line or second‐line treatment, including the 26 patients who received crizotinib after the failure of standard chemotherapy, are shown in Table 2.

**Table 2:** Demographic and clinicopathologic characteristics of patients who received crizotinib as first‐line or second‐line treatment

Characteristic	Total *N *= 56*n* (%)	First‐line crizotinib* N *= 30*n* (%)	Second‐line crizotinib* N *= 26*n* (%)	*P*‐Value
Age, years				
Mean	55	58	52	
Range	32–83	37–83	32–72	
Age distribution				
<60 years	37 (66)	17 (57)	20 (77)	0.159
≥60 years	19 (34)	13 (43)	6 (23)	
Gender				
Male	28 (50)	15 (50)	13 (50)	1.000
Female	28 (50)	15 (50)	13 (50)	
ECOG PS				
0	13 (23)	8 (27)	5 (19)	0.637
1	42 (75)	21 (70)	21 (81)	
2	1 (2)	1 (3)	0 (0)	
Radical surgery history				
Yes	10 (18)	7 (23)	3 (12)	0.310
No	46 (82)	23 (77)	23 (88)	
Smoking history				
Never	42 (75)	23 (77)	19 (73)	0.530
≤10 pack‐year	6 (11)	1 (3)	3 (12)	
>10 pack‐year	8 (14)	6 (20)	4 (15)	
Clinical stage				
IIIB	5 (9)	1 (3)	4 (15)	0.172
IV	51 (91)	29 (97)	22 (85)	

ECOG PS, Eastern cooperative oncology group performance status.

### Efficacy comparison of first‐line crizotinib and first‐line standard chemotherapy

The objective response rate (ORR) (CR and PR) of the 30 patients who received first‐line crizotinib was significantly higher than those who received first‐line chemotherapy (73.3% [95% CI: 57.5–89.1%] vs. 36.0% [95% CI: 22.7–49.3%], *P *= 0.002). The DCR (CR, PR, and SD) of crizotinib and chemotherapy groups was 93.3% (95% CI: 84.4–100%) and 78.0% (95% CI: 66.5–89.5%), respectively, (*P *= 0.116) (Table 3).

**Table 3:** Response to treatment according to RECIST

Response	Crizotinib N = 56	First‐line Crizotinib N = 30	First‐line Chemotherapy N = 50	Second‐line Crizotinib N = 26
CR, *n* (%)	1 (1.8)	1 (3.3)	0	0
PR, *n* (%)	38 (67.9)	21 (70.0)	18 (36.0)	17 (65.4)
SD, *n* (%)	13 (23.2)	6 (20.0)	21 (42.0)	7 (26.9)
PD, *n* (%)	4 (7.1)	2 (6.7)	11 (22.0)	2 (7.7)
ORR, %	69.6	73.3	36.0	65.4
DCR, %	92.9	93.3	78.0	92.3

CR, complete response; PR, partial response; SD, stable disease; PD, progressive disease; ORR, objective response rate; DCR, disease control rate.

**Figure 2:**
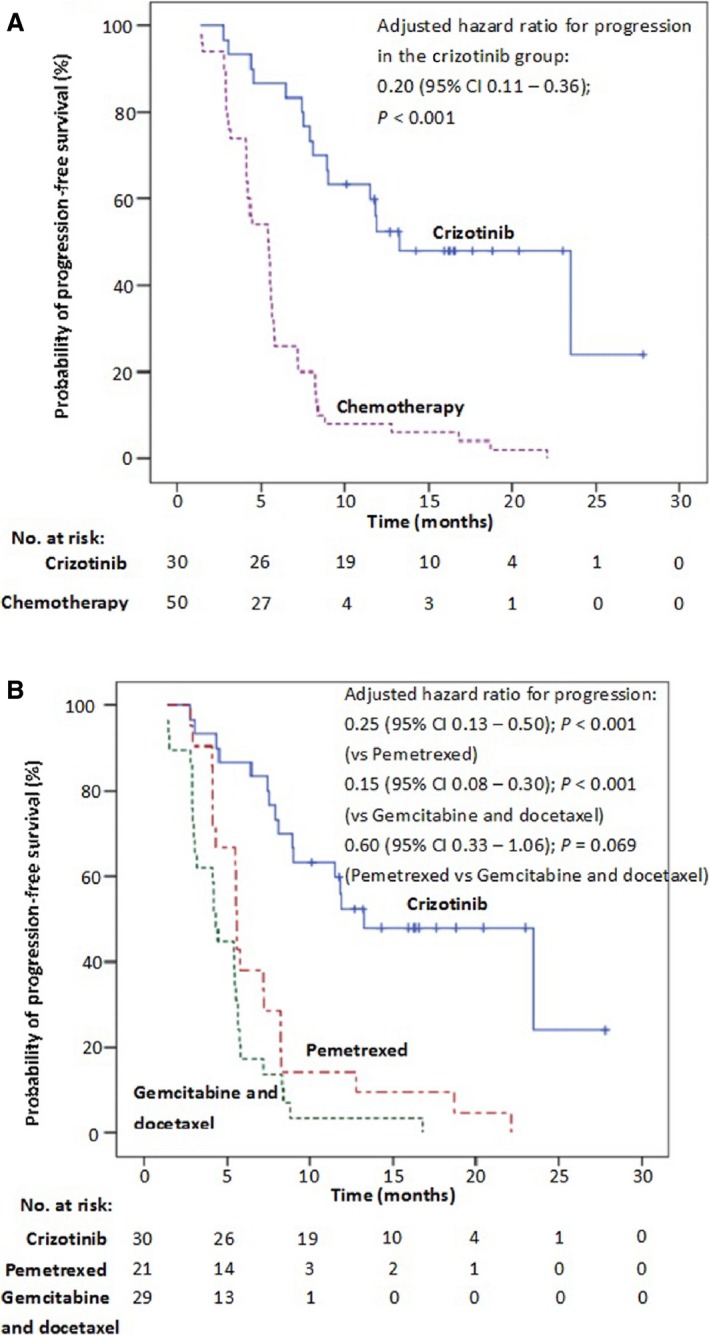
(A) Kaplan–Meier curves of progression‐free survival (PFS) with first‐line crizotinib and first‐line standard chemotherapy (adjusted hazard ratio for progression or death in the crizotinib group, 0.20, 95% CI: 0.11–0.36; *P *< 0.001). (B) Kaplan–Meier curves of (PFS) with first‐line crizotinib and first‐line pemetrexed plus platinum and docetaxel or gemcitabine plus platinum chemotherapy regimens (adjusted hazard ratio for progression or death in the crizotinib group, 0.25, 95% CI: 0.13–0.50; *P *< 0.001 vs. pemetrexed; 0.15, 95% CI: 0.08–0.30; *P *< 0.001 vs. gemcitabine and docetaxel; 0.60, 95% CI: 0.33–1.06; *P *= 0.069 pemetrexed vs. gemcitabine and docetaxel). Tick marks represent censored observations.

The median PFS was 13.3 months (95% CI: 6.5–20.0 months) in patients who received first‐line crizotinib as compared with 5.4 months (95% CI: 4.4–6.5 months) in patients who received first‐line standard chemotherapy (adjusted hazard ratio for progression or death with crizotinib, 0.20; 95% CI: 0.11–0.36; *P *< 0.001) (Fig. 2A). In subgroup analyses, there was a significant PFS benefit with crizotinib in comparison with both pemetrexed plus platinum (adjusted hazard ratio for progression or death with crizotinib, 0.25; 95% CI: 0.13–0.50; *P *< 0.001) and gemcitabine or docetaxel plus platinum (adjusted hazard ratio for progression or death with crizotinib, 0.15; 95% CI: 0.08–0.30; *P *< 0.001) (Fig. 2B). However, there was no statistically significant difference in PFS between patients who received pemetrexed plus platinum and those received gemcitabine or docetaxel plus platinum (5.6 months, 95% CI: 5.5–5.7 months vs. 4.3 months, 95% CI: 4.0–4.7 months; *P *= 0.069, log‐rank test).

### Efficacy comparison between first‐line crizotinib and second‐line crizotinib after disease progression on first‐line chemotherapy

Of the 26 patients who received second‐line crizotinib after the occurrence of PD on first‐line chemotherapy, the ORR and DCR was 65.4% (95% CI: 47.1–83.7%) and 92.3% (95% CI: 82.1–100%), respectively. No statistical differences were found between first‐line and second‐line crizotinib treatment with respect to ORR and DCR (*P *= 0.570 and 1.000, respectively) (Table 3).

**Figure 3:**
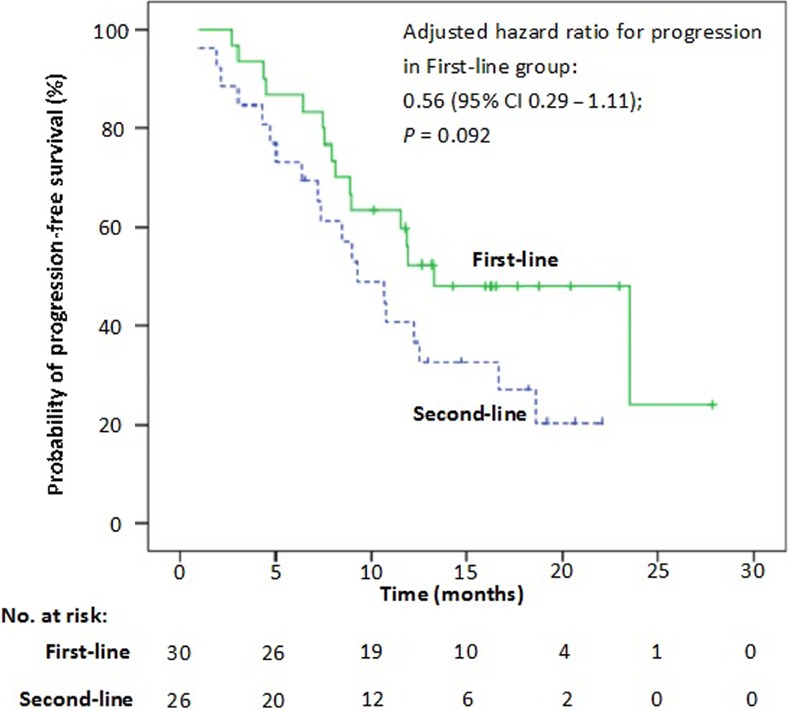
Kaplan–Meier curves of progression‐free survival (PFS) with first‐line and second‐line crizotinib therapy (adjusted hazard ratio for progression or death, 0.56, 95% CI: 0.29–1.11; *P *= 0.092).

**Figure 4:**
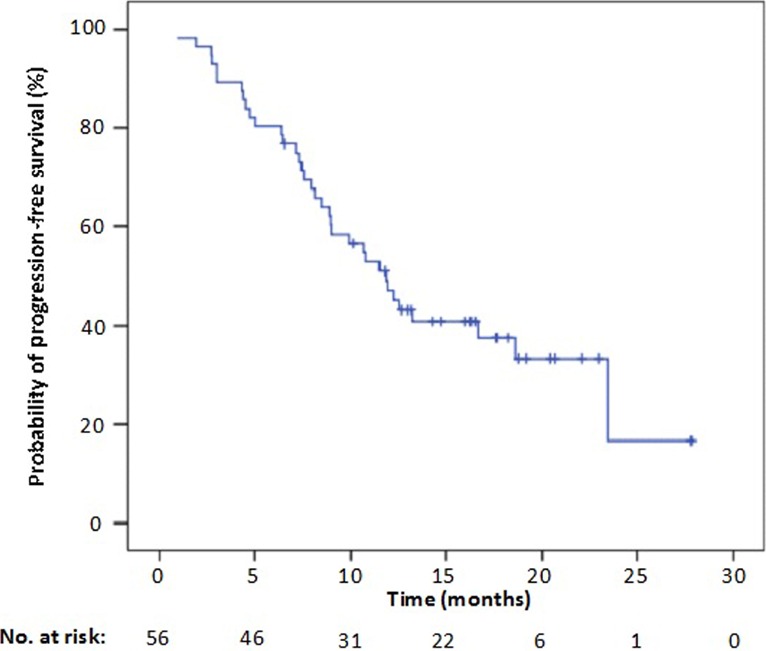
Kaplan–Meier curves of progression‐free survival (PFS) with crizotinib as either first‐line or second‐line treatment.

The median PFS in patients who received second‐line crizotinib was 9.9 months (95% CI: 6.4–13.4 months). There was no statistically significant difference in PFS between first‐line and second‐line crizotinib treatment (adjusted hazard ratio for progression, 0.56; 95% CI: 0.29–1.11; *P *= 0.092) (Fig. 3). The pooled median PFS of all 56 patients who received crizotinib as either first‐ or second‐line treatments was 11.8 months (95% CI: 9.3–14.4 months) (Fig. 4) and the ORR and DCR were 69.6% (95% CI: 57.5–81.6%) and 92.9% (95% CI: 86.2–99.6%), respectively (Table 3).

### Tolerability

Seventy‐five of the 80 treated patients (93.6%) experienced treatment‐related adverse events (Table 4). The most common adverse events with crizotinib were visual disturbances, which were most frequently described as trails of light following objects moving relative to the observer, and were noticed particularly during changes in ambient lighting from dark to light. In addition, gastrointestinal disturbances, including nausea, vomiting, diarrhea, and constipation were also frequent treatment‐related adverse events with crizotinib. However, most of these reactions were mild and transient, and often improved with an increasing duration of treatment on crizotinib. Grade 3 or 4 adverse events occurred in 8 of the 56 patients (14.2%) treated with crizotinib. Grade 3 elevations in alanine aminotransferase (ALT) and aspartate aminotransferase (AST) were recorded in two patients (3.6%), while five (8.9%) experienced grade 3 vomiting, and one patient had an acute pulmonary embolism, which led to death. Three patients in our study reduced the dose of crizotinib due to vomiting. Two patients reduced the dose of crizotinib as they experienced liver damage (ALT or AST elevation).

**Table 4:** Treatment‐related adverse events in the 80 patients

Adverse event	Crizotinib (first‐ or second‐line patients) (*N *= 56)		Chemotherapy (only first‐line patients) (*N *= 50)	
	Any grade^a^		Any grade^a^	
	*n*	%	*n*	%
Visual disturbance	29	52	2	4
Diarrhea	24	43	10	20
Nausea	21	38	24	48
Vomiting	16	29	11	22
Constipation	9	16	19	38
Decreased appetite	9	16	22	44
ALT elevation	9	16	6	12
Edema	6	11	2	4
Fatigue	6	11	28	56
AST elevation	5	9	5	10
Rash	4	7	6	12
Dizziness	3	5	7	14
Alopecia	2	4	11	22
Adverse event	Grade 3 to 4		Grade 3 to 4	
	*n*	%	*n*	%
Vomiting	5	9	2	4
ALT elevation	2	4	1	2
AST elevation	2	4	1	2
Pulmonary embolism	1	2	0	0
Fatigue	0	0	2	4

AE, adverse event; ALT, alanine aminotransferase; AST, aspartate aminotransferase.

a These adverse events occurred in at least 10% of the patients in either treatment group.

Twenty‐eight (56%) of the 50 patients who received first‐line standard chemotherapy experienced fatigue, which was the most common adverse event in this group. Gastrointestinal disturbances were also common treatment‐related adverse events in these patients, but these manifestations were able to be relieved by symptomatic therapies.

## Discussion

The findings of this study suggest that first‐line crizotinib treatment resulted in significantly higher response rates and a longer PFS in comparison with first‐line standard chemotherapy in Chinese patients with *ALK*‐positive, advanced lung adenocarcinoma. Crizotinib also showed promising efficacy when used as a second‐line treatment after the failure of first‐line chemotherapy in these patients.

A recent open‐label, phase 3 trial suggested that first‐line crizotinib was superior to standard pemetrexed plus platinum chemotherapy in patients with previously untreated, advanced, *ALK*‐positive non‐squamous NSCLC 14. At WCLC 2013, a study showed data on the activity of crizotinib within PROFILE 1007 on Asiatics 15. In this study, PFS was significantly longer with crizotinib than with chemotherapy (median 8.1 vs. 2.8 months; hazard ratio, 0.53; *P *= 0.003), and the ORR on crizotinib (75%) was significantly higher than on chemotherapy (22%; *P *< 0.0001) 15. In the present study, we found a higher ORR and significant PFS benefit of first‐line crizotinib versus standard chemotherapy in *ALK*‐positive Chinese lung adenocarcinoma patients, which was consistent with previous reports. Overall survival (OS) data in the present study were not mature, as only 10 patients (12.5%) had died at the cutoff date. In previous studies, OS did not significantly differ between crizotinib and standard chemotherapy in chemotherapy‐naïve patients with non‐squamous NSCLC 14.

Platinum‐based chemotherapy is one of the regimens traditionally used for advanced NSCLC. Existing studies have indicated that pemetrexed plus platinum may be superior to gemcitabine plus platinum in previously untreated, non‐squamous NSCLC 16. Previous studies conducted in *ALK*‐positive NSCLC revealed that pemetrexed‐based chemotherapy was more efficient than other regimens 13, which suggested that patients with *ALK*‐positive NSCLC may have a higher response rate with pemetrexed than does the general population with NSCLC 13, 17, 18. In this study, we compared the PFS of patients with lung adenocarcinoma who received pemetrexed plus platinum with those who received gemcitabine or docetaxel plus platinum, and found no statistical difference of PFS between these regimens. A potential limitation of this comparison was that the sample size of pemetrexed‐based and gemcitabine‐ or decetaxel‐based group was relative small compared with previously studies, and a longer trend of pemetrexed‐based chemotherapy for PFS could be seen from Figure 2B albeit there was no statistical differences between them (*P *= 0.069). This study also indicated that crizotinib has promising efficacy in *ALK*‐positive Chinese lung adenocarcinoma patients who received it as a second‐line treatment after PD had occurred on first‐line chemotherapy, with a median PFS of about 10 months and an ORR of more than 60% in these patients. This suggests that *ALK*‐positive lung adenocarcinoma patients who are assessed as having PD on first‐line chemotherapy might continue to receive and benefit from crizotinib. In addition, we found that the tolerability profiles of crizotinib and chemotherapy differed markedly, and the adverse effects of crizotinib were generally mild and transient in comparison with those of standard chemotherapy. Visual disturbances and mild gastrointestinal reactions were the most common adverse events noted with crizotinib, whereas fatigue and gastrointestinal disturbances occurred frequently in patients who received standard chemotherapy and these adverse effects often needed to be alleviated with symptomatic treatment. Additionally, patients who received crizotinib only required regular follow‐ups after the drug's initial administration, whereas those who received standard chemotherapy required hospitalization which affected their quality‐of‐life (QoL) to some extent. Although QoL data before and after treatment were not collected in this study (because of its retrospective nature), previous studies have indicated that there is a significantly greater overall improvement from baseline in global QoL among patients who received crizotinib than among those who received chemotherapy 13, 14. In the analysis of Asiatics patients in PROFILE 1007, crizotinib treatment was associated with a significantly longer time to disappear (TTD) in lung cancer symptoms compared with chemotherapy 15. Additionally, significantly greater improvement from baseline was observed with crizotinib for global QoL 15. Thus, initiating crizotinib as first‐line treatment may also improve QoL in addition to the benefits that arise from its direct *ALK* inhibitory effect in *ALK*‐positive NSCLC.

As yet, differences in efficacy between first‐line and second‐line crizotinib treatment have not been extensively investigated. In the present study, there were no statistically significant differences in ORR, DCR and PFS between patients who received first‐line and second‐line crizotinib treatment. A recent study reported similar efficacies of epidermal growth factor receptor tyrosine kinase inhibitors (*EGFR*‐TKIs) in patients with *EGFR* mutation‐positive adenocarcinoma in terms of ORR, PFS, and OS, irrespective of the timing of treatment 19. As targeted therapies are currently the preferred first‐line treatments in patients with mutation‐positive tumors, this finding indicates that sequential treatment with targeted therapies could be a reasonable option when the mutation status cannot be quickly determined.

In the present study, 17 patients who received crizotinib as first‐ or second‐line treatment continued crizotinib therapy after PD had been defined because we believed that they would acquire clinical benefit from the drug. Targeted molecular therapies are increasingly being continued beyond the occurrence of PD, since many patients harboring an sensitive mutation could acquire continuously clinical benefits after RECIST‐defined PD 20, 21. Recently, a retrospective study indicated that continuing *ALK* inhibition with crizotinib after PD has occurred may provide a survival benefit to patients with advanced, *ALK*‐positive NSCLC 20. Thus, RECIST‐defined PD may not be a suitable standard for discontinuation of targeted therapies. Whether long‐term clinical benefit is derived from crizotinib will need to be reassessed for patients with RECIST‐defined PD.

This study has some limitations. Firstly, it was a retrospective, single‐center study with a relatively small sample size, and its findings therefore need to be confirmed by subsequent prospective, multicenter studies with larger sample sizes. Secondly, the PFS data for patients who received second‐line crizotinib after the failure of first‐line chemotherapy should be interpreted with caution, as the patients who received second‐line crizotinib had a relatively shorter follow‐up time in comparison with those who received first‐line crizotinib.

In conclusion, there was a significant PFS benefit of first‐line crizotinib versus first‐line standard chemotherapy among Chinese patients with *ALK*‐positive lung adenocarcinoma. Additionally, crizotinib showed promising efficacy in patients who received the drug as a second‐line treatment after PD had occurred on first‐line chemotherapy.

## Conflict of Interest

The authors declare no conflicts of interest.
